# Control of electrical conductivity of highly stacked zinc oxide nanocrystals by ultraviolet treatment

**DOI:** 10.1038/s41598-019-42102-3

**Published:** 2019-04-18

**Authors:** Wooje Han, Jiwan Kim, Hyung-Ho Park

**Affiliations:** 1 Department of Materials Science and Engineering, Yonsei University, 50 Yonsei-ro, Seodaemun-gu, Seoul, 03722 Republic of Korea; 2 Department of Advanced Materials Engineering, Kyonggi University, 154-42 Gwanggyosan-ro, Suwon-si, 16227 Gyeonggi-do Republic of Korea

**Keywords:** Nanoparticles, Nanoparticles

## Abstract

Zinc oxide (ZnO) nanocrystals (NCs) were synthesized using a modified sol-gel method. Ultraviolet (UV) treatment was performed under various atmospheres on the highly stacked ZnO NCs. The prepared NCs were characterized using Fourier transform infrared spectroscopy, scanning electron microscopy, X-ray diffraction, photoluminescence spectroscopy, and atomic force microscopy to investigate their structural, electrical, and electrochemical properties. Through these analyses, the effect of the UV treatment on the chemical and electrical characteristics of ZnO NCs was established. According to the analyses, the organic ligands in the NCs were decomposed, and the particles were densified. The mobility of UV-treated ZnO NCs thin films increased to 1.4 cm^2^/Vs, almost 2 orders higher than the UV untreated ZnO thin films. It was confirmed that the recombination from oxygen vacancies of ZnO could be controlled by UV irradiation. As decreased oxygen vacancies, the band gap of ZnO NCs was increased from 3.2 eV to 3.27 eV.

## Introduction

Zinc oxide (ZnO) has several favorable properties, including high electron mobility, a wide bandgap, and strong room-temperature luminescence^1–4^. These properties are valuable in numerous applications, including energy-saving or heat-protecting windows and electronics, such as thin-film transistors, light-emitting diodes, and transparent electrodes in liquid-crystal displays^5–9^. ZnO has a direct band gap of 3.3 eV at room temperature with a large exciton binding energy of 60 meV^10^. The native doping state of ZnO is n-type owing to oxygen vacancies or zinc interstitials^11,12^. Additionally, good thermal stability and high electron mobility of ZnO make it a strong candidate for the electron transport layer in quantum dot light-emitting device (QLED). When ZnO is applied to QLED, not only high mobility but also balances of the electrons and the holes in the emission region should be considered^13^. Due to the quantum confinement effect of nanometer scale ZnO, size-dependent optical absorption is a valuable tool for studying synthesis and growth of ZnO^14^.

Commonly oxide nanocrystals (NCs) have been synthesized using the hydrothermal and sol-gel method^15,16^. Unlike the hydrothermal method under high pressure condition, the sol-gel method can synthesize oxide using metal alkoxide precursors under ambient condition^17^. A sol-gel preparation method was demonstrated as simple route to prepare small nanosized oxide NCs^18–20^.

The capping ligand of NCs is both essential and disadvantageous for applying NCs. The capping ligands are necessary to control oxide NCs, but they can degrade properties of materials^21^. In particular, the capping ligands of NCs act as carrier trapping site or an Auger recombination site, degrading an electrical display devices^22^. Studies on replacing the ligands with shorter ones and controlling the ligand length using metal chalcogenides and monovalent inorganic ligands have been performed to overcome the disadvantages of capping ligands^23^. However, these treatments have limitations. They are vulnerable to the oxidation of the device own working heat and to the loss of the dispersibility of the NCs, which introduces disadvantages for applying the device. Ultraviolet (UV) treatment was suggested to overcome the ligand exchanging treatment. UV irradiation can be used to remove ligands of NCs^24^. This can yield highly stacked NCs and lead to an increase in the emission efficiency due to the eliminated trap site on the surface. The electrical mobility could be also controllable using this UV irradiation. Usually, heavy element doping or ligand exchange of metal oxide has been studied to control the mobility of oxides^13^. It is possible to control the electron mobility of ZnO through only UV irradiation. In the present study, ZnO NCs were prepared and shown to have highly stacked properties through thin film processing.

## Results and Discussion

**Figure 1:**
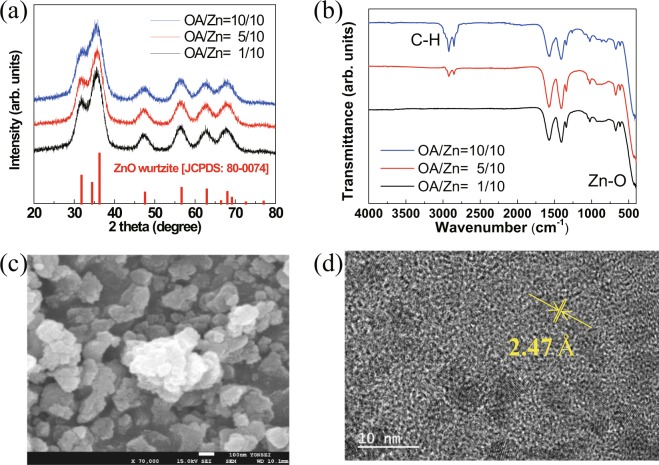
(**a**) XRD pattern; (**b**) FT-IR spectra of ZnO NCs with various concentrations of OA ligands; (**c**) SEM image; (**d**) TEM image of ZnO NCs with OA/Zn = 10/10.

The ZnO NCs were synthesized by the Spanhel and Anderson method^19^. Figure 1(a) shows the XRD patterns of the ZnO NCs. The ZnO NCs were identified as the wurtzite phase by comparison with the reference JCPDS card number 80-0074^25^. The calculated crystalline sizes of the ZnO NCs were 4.2, 3.8, and 3.5 nm for oleylamine (OA)/Zn ratios of 1/10, 5/10, and 10/10, respectively. The sizes of the ZnO NCs were calculated via the Scherrer equation using the full width at half maximum from XRD^26^. The smallest size of ZnO was confirmed by OA/Zn = 10/10. To confirm the relationship between the OA/Zn ratio and the crystalline size of ZnO NCs, FT-IR measurements of as-synthesized ZnO NCs were performed, and the results are shown in Fig. 1(b). The Zn-O bonding absorption existed at 470 cm^−1^ ^27^. The absorption around 1,500 cm^−1^ was indicated by the acetate group in the ZnO precursor. ZnO NCs were synthesized using zinc acetate. The acetate functional group remained on the surface of the NCs. The absorption of 1,576 and 1,403 cm^−1^ indicated the symmetrical and asymmetrical stretching modes, respectively, of the carboxylate groups of acetate^28^. These results were generally obtained in ZnO using acetate precursors^29^. The concentration of OA was changed to control the size of the NCs, and the C-H absorption (2,800 cm^−1^) results were changed with the increasing concentration^30^. As the ligand concentration increased, the long carbon of OA affected the formation of the ZnO crystal structure, and the CH_2_ absorption (2,800 cm^−1^) increased. In addition, the absorptions of the 1,500 cm^−1^ region containing the acetate group decreased. These results confirm that the surface termination of ZnO NCs was changed from acetate to OA. Together with the XRD results, it was confirmed that the size of the NCs could be reduced by the OA ligand. Figures 1(c,d) show SEM and TEM images of ZnO with the smallest size of NCs, respectively. The NC size was confirmed as approximately 3.5 nm, and the real lattice distance was 2.47 Å^25^. The 2.47 Å lattice distance of ZnO corresponds to the (101) XRD diffraction peak with the highest intensity at 36°. Additionally, all the nanocrystals showed that the particles are well dispersed, without an agglomeration (Fig. S1).

**Figure 2:**
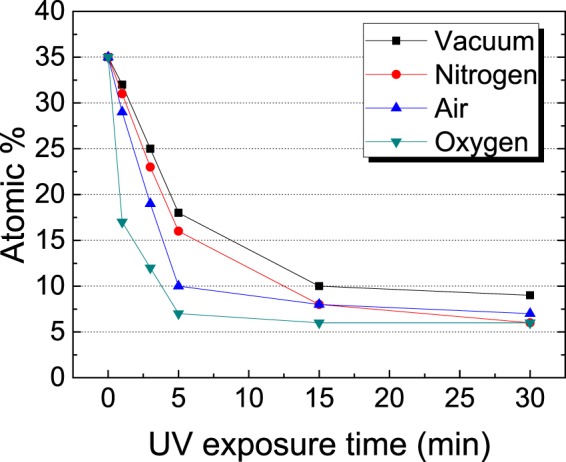
Carbon atomic percent of UV-treated ZnO NC thin films under various atmospheres.

The UV irradiation experiments were performed using spin-coated thin-film ZnO NCs. Various atmospheres were employed, including an inert atmosphere and reactive conditions involving vacuum, nitrogen, air, and oxygen. Figure 2 presents the carbon compositional change over time for each circumstance. The carbon compositional change was monitored using XPS (detailed data are presented in Fig. S2 of the Supplementary Information). The functional groups and C-C bonding of the ligand were decomposed by UV exposure under any atmospheric condition. After the UV exposure, approximately less than 10% of carbon remained owing to the fractured carbon that stayed out between NCs. Table 1 shows the compositional atomic percent data before (pristine) and after 30 min of UV treatment. The ratio of Zn to O was maintained after the UV treatment. ZnO is a material with UV resistance and did not undergo a change in morphology or composition. On the other hand, the ligand was an organic material, and the UV irradiation could form radicals in the functional group and the carbon backbone. The radical could have caused the decomposition. The UV irradiation showed that the ratio of Zn-O was maintained while the amount of carbon was decreased with time in all circumstances. The specific data of EDX and FT-IR according to the UV irradiation time in each atmosphere are presented in Tables S1–S4 and Figs S4–S7 of the Supplementary Information. The C-C bond of ligand is easily excited by ultraviolet irradiation. The generated radicals from excitation can break carbon ligand chains. These radicals induce ligand fracture^31,32^. The decomposition of the ligand was faster in air and oxygen atmosphere. This is because of the ozonation of oxygen, as follows^33^:

$${{\rm{O}}}_{{\rm{2}}}+{hv}({\rm{UV}})\to {\rm{2O}}\cdot $$

$${\rm{O}}\,\cdot +{{\rm{O}}}_{{\rm{2}}}\to {{\rm{O}}}_{{\rm{3}}}$$

**Table 1:** Composition of ZnO NC thin films before and after UV treatment under various atmospheres.

Atmosphere	Pristine	Vacuum	N_2_	Air	O_2_
C at%	35	9	7	7	6
O at%	32	46	46	46	45
Zn at%	33	47	47	47	48

**Figure 3:**
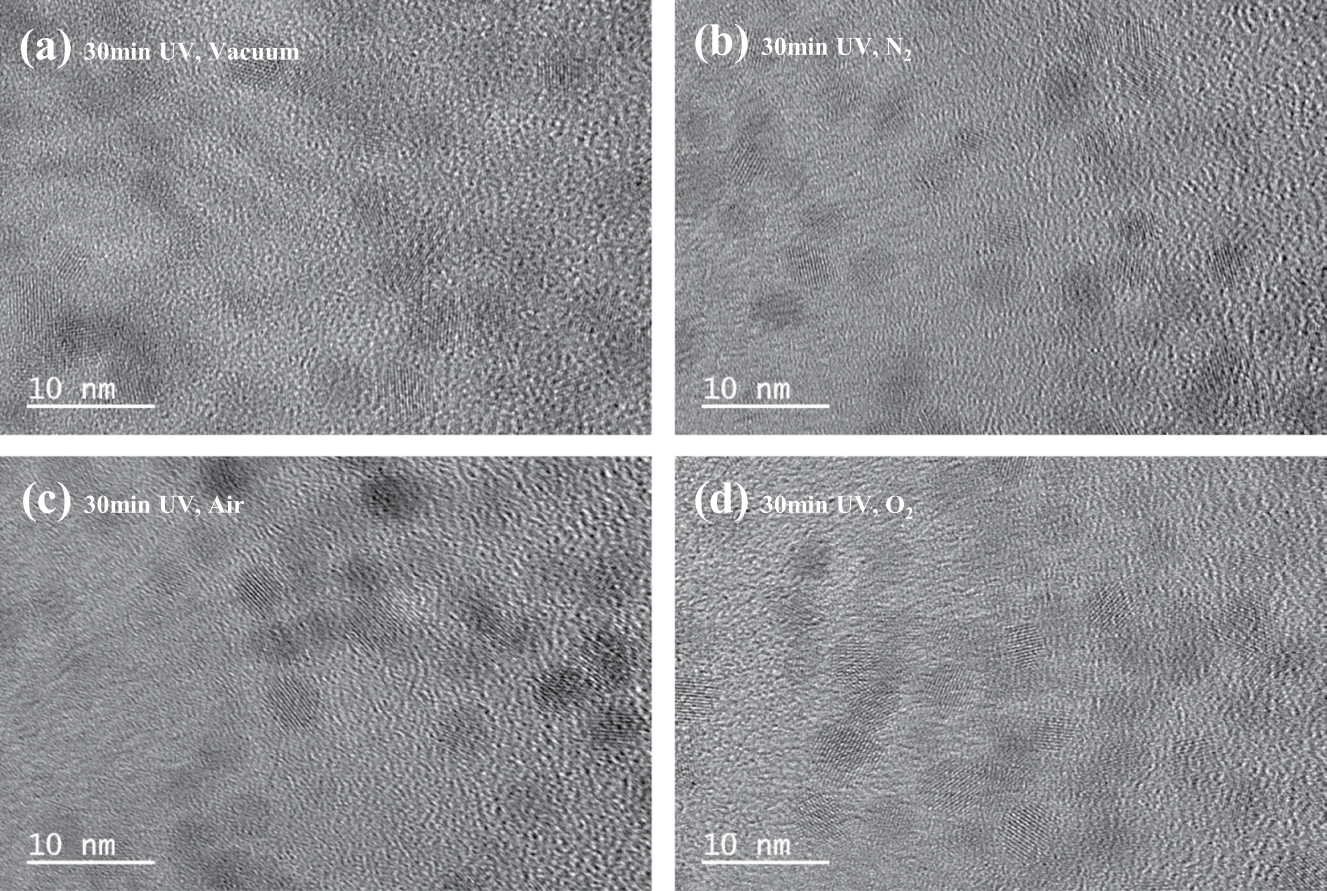
TEM images of UV-treated ZnO NC thin films under various atmospheres.

An ozone was generated by the reaction between UV rays and oxygen in air. In the oxygen atmosphere, more ozone could be generated by the UV rays. The abundant oxygen atmosphere confirmed that the ligand degradation was drastically changed compared with the carbon composition. TEM images showed ZnO NCs treated for 30 min in various atmosphere (Fig. 3). The longest-exposed NCs were also confirmed to have no crystal-size change after the UV irradiation. No additional agglomeration or morphology changes were confirmed by the TEM images.

**Figure 4:**
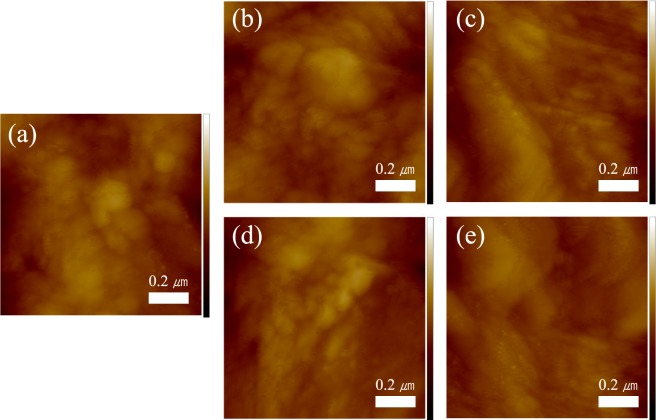
(**a**) AFM images of a pristine ZnO NC thin film and ZnO NC thin films UV-treated for 30 min under various atmospheres: (**b**) vacuum; (**c**) nitrogen; (**d**) air; and (**e**) oxygen (scale ranges from 5 nm to -5 nm, corresponding to white to black, respectively).

The AFM images of ZnO NC thin films before and after UV irradiation were given in Fig. 4. Figure 4(a) shows an AFM image of pristine ZnO NC thin film. The root-mean-square (RMS) roughness and thickness obtained from AFM and SEM cross section images are summarized in Table 2. The thickness and RMS roughness of the films were reduced owing to the ligand decomposition of thin film NCs and a densification of thin films was induced. The flatness of the film in the oxygen atmosphere could be controlled to 66% of that of the pristine thin film because higher removal of the carbon fracture yields a higher density and lower roughness. The thin film was formed by agglomerated particles as described in Fig. S10. The void that comes from ligand decompose of carbon chains in oleylamine was controlled by densification that result of rearrangement of the nanocrystals.

**Table 2:** RMS roughness and thickness of ZnO NC thin films.

Atmosphere	Pristine	Vacuum	N_2_	Air	O_2_
RMS roughness	2.52 nm	2.03 nm	2.1 nm	1.62 nm	1.71 nm
Thickness	45 nm	32 nm	35 nm	31 nm	30 nm

**Figure 5:**
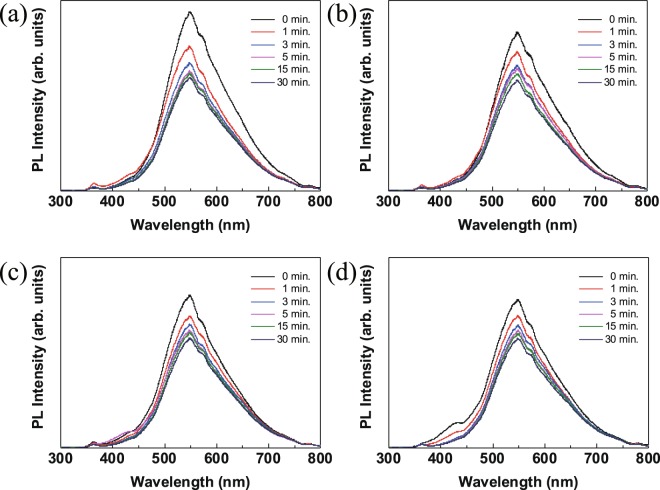
PL spectra of UV-treated ZnO NC thin films: (**a**) vacuum; (**b**) nitrogen; (**c**) air; and (**d**) oxygen.

**Figure 6:**
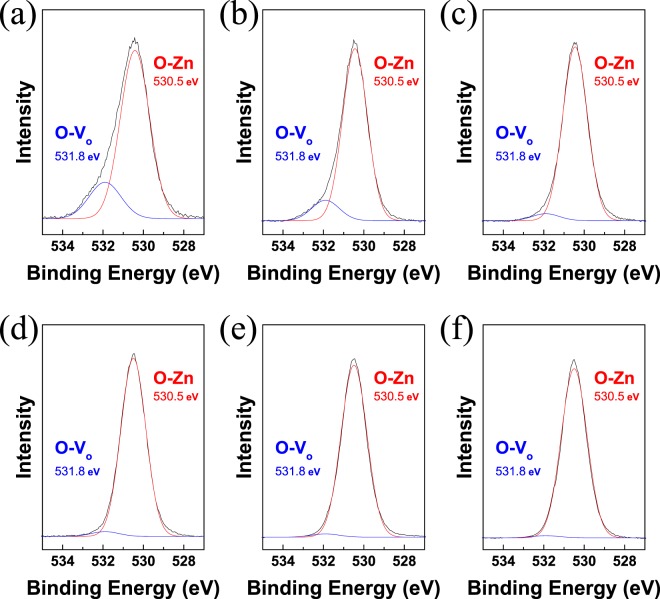
O 1 s XPS spectra of UV-treated ZnO NC under oxygen atmosphere: (**a**) 0 min., (b) 1 min., (**c**) 3 min., (**d**) 5 min., (**e**) 15 min., and (**f**) 30 min.

The photoluminescence (PL) spectra of the ZnO NCs thin films after various atmospheric UV treatment are shown in Fig. 5. The 360 nm emission is near-band-edge transition^34^. An absorption band at around 425 nm corresponds to Zn interstitials shallow donors in ZnO NCs^35^. Especially a sharp decrease was observed in the case of UV treatment under O_2_ atmosphere due to effective filling of oxygen vacancy from strong oxidant of ozone^36^. The PL intensity of 550 nm (green) region was decreased in all the thin films by increasing the irradiation time. The 550 nm region corresponds to the surface recombination of emission-related singly ionized oxygen vacancies^37^. The controlled oxygen vacancies of ZnO were confirmed by the changes in the PL intensity. In Fig. 6, O1s XPS analysis was performed to determine whether the ZnO oxygen vacancies could be controlled via UV irradiation. The oxygen of ZnO had various bonds in the O1s region. The O-Zn bond in the ZnO lattice without an oxygen vacancy was appeared at 530.5 eV and O-Zn bonds at 531.8 eV region were related to oxygen vacancies in the lattice (O-V_o_)^38^. The Zn-O bonds without oxygen vacancies were increased by UV irradiation. With UV irradiation, the peak intensities of O-Zn and O-V_o_ were increased and decreased, respectively. UV irradiation arranged the oxygen to passivate the vacancies of the lattice in the NCs and controlled the presence of vacancies in the lattice. Further, the intensity change of the rapid O-V_o_ was confirmed by 1 min UV treatment in an oxygen atmosphere. Passivation of vacancies could be occurred by surface oxygen molecules in surface of NCs^39,40^. The surface oxygen can generate the O* radicals during UV treatment. The O* radicals can act as oxygen vacancies controller. The passivation of oxygen vacancies was found to be accelerated in oxygen atmosphere^41^ (Fig. S3).

**Figure 7:**
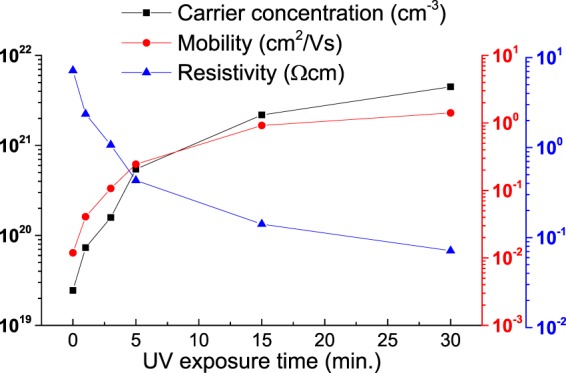
Electrical properties of UV-treated ZnO NC thin films under oxygen atmosphere.

The electrical properties of UV treated ZnO NCs thin films in oxygen atmosphere was given at Fig. 7. The increase of carrier concentration and mobility and decrease of resistivity were confirmed using Hall effect measurement system. The NCs thin films with more ligands showed lower mobility because the ligands on NCs surface interrupted the current flow of NCs thin films^42^. The mobility of UV treated ZnO NCs thin films was increased to 1.4 cm^2^/Vs from 1.2 × 10^−2^ cm^2^/Vs. The high mobility means that the carrier scattering was also reduced because of enhanced particles-contact. The decrease of oxygen vacancy could also make an increase of mobility^43^.

This is well supported from the PL results given in Fig. 5. In some cases of thin filming of transparent conducing oxides, UV treatment is used to increase the mobility^44^. The effect of densification of NCs thin film by UV treatment was observed while elimination of ligands. The trapping carriers on NCs was reduced by elimination of ligands. Trapping carrier was related with controlling of oxygen vacancies. In addition, the carrier was increased due to the reduction of roughness and reduction of trapped carriers^45^. The electrical property of UV treatment was more affected by ligands than by ZnO.

**Figure 8:**
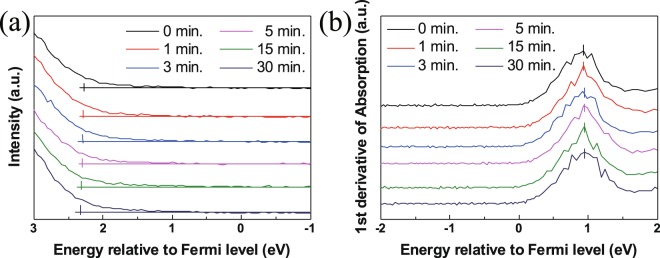
(**a**) Valence band spectrum; (**b**) first derivation value of the O 1 s NEXAFS of UV-treated ZnO NC thin films under oxygen atmosphere.

The resistivity changes of about 1 order are usually reported in the conventional UV treatment^46^, but in oxygen atmosphere (Fig. 7), almost 2 orders of difference was observed. The band structure of UV treated ZnO NCs thin films in oxygen atmosphere were evaluated with the valance band and O 1 s NEXAFS spectra as given in Fig. 8. Figure 8(a) shows the Fermi energy (E_F_) region using 90 eV photon energy. The energy differences of the valence band maximum (VBM) from Fermi level were 2.28, 2.29, 2.29, 2.3, 2.31 and 2.33 eV with increasing UV exposure time in oxygen atmosphere. Figure 8(b) shows the first derivation of O 1 s NEXAFS. The absorption spectra were calibrated using the binding energy of O 1 s core electron. The energy differences of the conduction band maximum (CBM) from Fermi level were 0.92, 0.92, 0.93, 0.93, 0.94 and 0.94 eV with increasing UV exposure time. Therefore, the band gap of UV treated ZnO NCs were 3.2, 3.21, 3.22, 3.23, 3.25 and 3.27 eV to increasing UV exposure time. The band gap widening about 0.07 eV was caused by a decrease of oxygen vacancies from the curing of delocalized states in the valance band. The separation of the non-localized oxygen vacancy state with valance band decreases the valance band level, resulting in a band gap widening^47^. This result also explains as a polarization effect resulted from the surface bonding between ZnO NCs and OA. Such fluctuations in energy gaps are likely to originate from polarization effects on the surface of the nanocrystals, which depend on the number and bonding direction of passivating ligands, i.e. absolute values of their static dipoles. The decreasing of ligand induces a reduced polarization effect. Therefore, band gap was opened by decreased polarization effect^48^. The shift is resulted from the size effect of ZnO NCs. A blue shift of ZnO NCs in near-band-edge transition region depending on the size decrease of NCs was reported by Yang *et al*.^49,50^. In this experiment, blue shift from 380 nm to 360 nm was observed due to the small size of ZnO NCs, smaller than 5 nm. There was neither size variation of ZnO NCs from the UV treatment and nor shift in PL absorption wavelength.

UV irradiation treatment caused a densification of thin films and a passivation of oxygen vacancies of NCs. However, UV treatment confirmed that the phenomena occurring on the surface of NCs are more effective when compared with crystal lattice. In addition, improvement of mobility and band gap opening were confirmed due to an elimination of ligand. The high flatness and high crystallinity could be considered as a benefit to NCs application^51^. It is possible to remove a defect site that can be generated by the presence of a ligand and to control a defect site existed in the crystal through the UV treatment as a surface treatment technique of NCs thin films. This treatment can also be applied to improvement of efficiency of balances between electrons and holes in the emission region in QLED. Auger recombination that occurs on the surface of NCs is responsible for efficiency roll-off (also known as droop) at high-driving currents in emission applications. It is also possible to increase the NC efficiency owing to densification. UV treatment can improve the charge balance within the NCs by impeding electron injection, which leads to higher efficiency and better roll-off behaviour in devices.

## Conclusion

ZnO NCs were synthesized at a low temperature and ZnO thin films were formed using spin coating. An elimination of NC ligands, and densification and planarization of thin films were obtained by UV irradiation. UV treatment also passivated the oxygen vacancies in the NCs. The band gap of ZnO NCs thin films after UV treatment for 30 min in oxygen atmosphere was changed from 3.2 to 3.27 eV due to decreased surface ligands and oxygen vacancies of NCs. The mobility of UV treated ZnO NCs thin films improved almost 2 orders as 1.4 cm^2^/Vs due to better particles contact. This UV irradiation technique can be used for a control of defects in NCs and for enhancing the interfacial properties through the densified stacking of NCs in the functional thin-film layer.

## Methods

Zinc acetate dihydrate (Zn(CH_3_COO)_2_·2H_2_O, ≥98%, Sigma-Aldrich, USA), ethanol (CH_3_CH_2_OH, 99.9%, Duksan, South Korea), oleylamine (OA, C_18_H_35_NH_2_, 70%, Sigma-Aldrich, USA), and lithium hydroxide hydrate (LiOH·H_2_O, ≥99.0%, Sigma-Aldrich, USA) were used as the starting zinc precursor, solvent, capping agent, and catalyst without any purification, respectively. Zinc acetate dihydrate and lithium hydroxide hydrate were dissolved in ethanol via ultrasonication. Oleylamine was introduced as a size controller of the NCs. The mixture solution was kept under vigorous stirring at room temperature. Later, the NCs were separated using a centrifuge, and the remaining organic materials and impurities were removed by washing several times with hexane and heptane. For centrifuging and washing of NCs, ethanol washing was also applied for several times for distribution of NCs and removal of water and impurity organic materials. The resulting ZnO NCs were dried at 50 °C for 12 h in an oven (a flowchart of the experiments is presented in the Supplementary Information as Fig. S9). The crystallinity of the ZnO NCs was estimated using X-ray diffraction (XRD, Ultima model, Rigaku, Japan) analysis with Cu-Kα radiation having a wavelength of 1.5418 Å at 2θ values in the range of 20° to 80°. The status of the surface ligands and ZnO NCs was confirmed using Fourier transform infrared spectroscopy (FT-IR, Perkin Elmer, USA) in the final product.

ZnO thin films were formed for UV treatment. The synthesized ZnO was formed as a thin film using spin casting. The thin films were densified via UV irradiation (365 nm, 1,200 W) under various atmospheres (a schematic of the experiments is presented as Fig. S10 in the Supplementary Information). A quartz box was applied for controlling the atmosphere under UV irradiation. The scanning electron microscopy (SEM, AIS-2000C, SERON, South Korea), transmission electron microscopy (TEM, JEOL-2100F, JEOL, Japan), and atomic force microscopy (AFM, MultiMode 8, Bruker, USA) were used to evaluate the distributions, morphologies, and roughness. Energy-dispersive X-ray spectroscopy (EDX, EDAX, Ametek, USA) was performed to determine the chemical composition. The carrier concentration of the films was obtained by the van der Pauw method at room temperature using a Hall effect measurement system (Ecopia, HMS3000, South Korea) with a direct current (I_DC_ = 10 mA) four probe method in a magnetic field up to 0.58 T. The chemical bonding state of ZnO NCs were obtained using photo emission spectroscopy and near-edge X-ray absorption fine structure (NEXAFS) analyses at the 4D and 10A2 beamlines of the Pohang Accelerator Laboratory. Gold was used as a reference for the energy calibration.

## Supplementary information

Control of electrical conductivity of highly stacked zinc oxide nanocrystals by ultraviolet treatment
