# Supplementary material for: Control of electrical conductivity of highly stacked zinc oxide nanocrystals by ultraviolet treatment
Source: Sci Rep. 2019 Apr 18;9:6244. doi: 10.1038/s41598-019-42102-3 (PMC6472384; doi:10.1038/s41598-019-42102-3)
Supplement: Supplementary file 1 — Control of electrical conductivity of highly stacked zinc oxide nanocrystals by ultraviolet treatment [file 41598_2019_42102_MOESM1_ESM.docx]

Correspondence and requests for materials should be addressed to H.H.P ([hhpark@yonsei.ac.kr](mailto:hhpark@yonsei.ac.kr))

Supplemental Information for

**Control of electrical conductivity of highly stacked zinc oxide nanocrystals by ultraviolet treatment**

Wooje Han^1^, Jiwan Kim^2^*, and Hyung-Ho Park^1^*

^1^Department of Materials Science and Engineering, Yonsei University, 50 Yonsei-ro, Seodaemun-gu, Seoul 03722, Republic of Korea
^2^Department of Advanced Materials Engineering, Kyonggi University, 154-42 Gwanggyosan-ro, Suwon-si, Gyeonggi-do, Republic of Korea

*Corresponding Authors: Jiwan Kim and Hyung-Ho Park

*e-mail: [jiwank@kyonggi.ac.kr](mailto:jiwank@kyonggi.ac.kr), [hhpark@yonsei.ac.kr](mailto:hhpark@yonsei.ac.kr)

**ZnO nanocrystals**


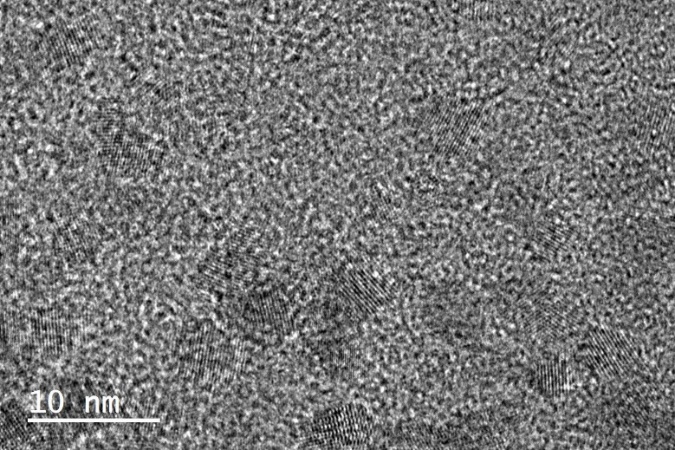

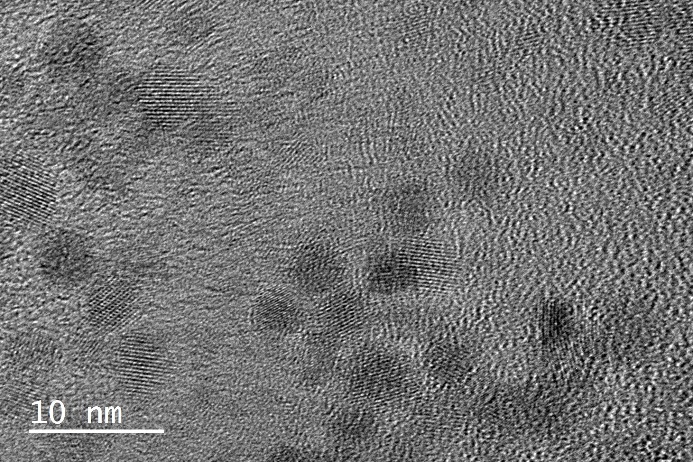

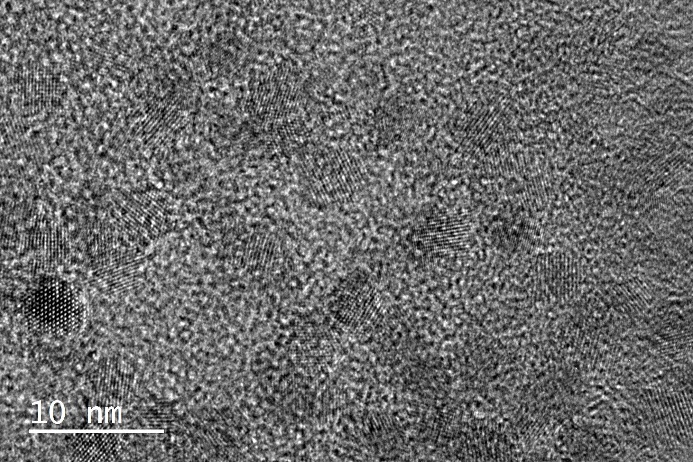


**(a) OA/Zn=1/10**

**(b) OA/Zn=5/10**

**(c) OA/Zn=10/10**

Figure S1. TEM images of ZnO NC with various OA/Zn component (OA/Zn = 1/10, 5/10 and 10/10).


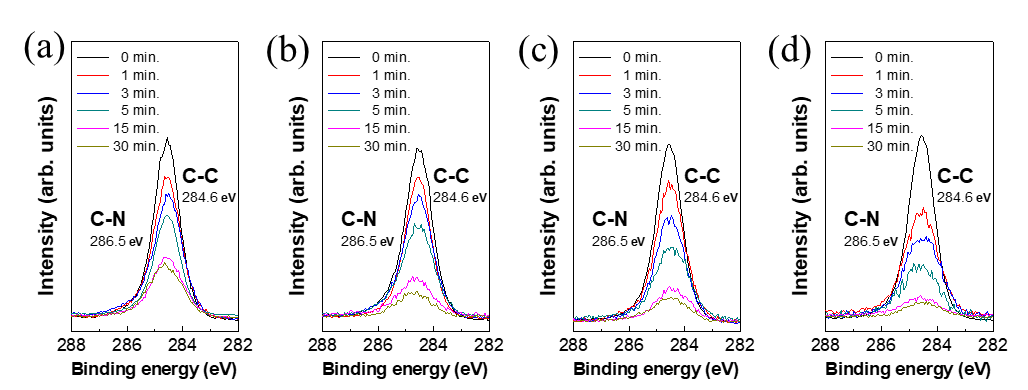
**UV irradiation of thin-film ZnO nanocrystals**

Figure S2. C1s XPS spectra of UV-treated thin-film ZnO NCs: (a) vacuum; (b) nitrogen; (c) air; and (d) oxygen.

The C-C bonds were observed at 284.6 eV. The C-N bonds were represented at 286.5 eV from the OA ligand [S1]. The decomposition of the ligands under UV irradiation was confirmed. The functional groups and C-C bonding of the ligand were decomposed by the UV exposure.

(a)

(b)

(c)

(d)

Figure S3. O1s XPS spectra of UV-treated ZnO NC thin films: (a) vacuum; (b) nitrogen; (c) air; and (d) oxygen.

**UV irradiation under vacuum atmosphere**

(a)

(b)

(c)

Figure S4. (a) Narrow FT-IR spectra of UV treated thin-film ZnO NCs under a vacuum atmosphere, EDX spectrum of UV-treated thin-film ZnO NCs under a vacuum atmosphere: (b) 0 min and (c) 30 min.

Figure S4(a) presents Fourier transform infrared spectroscopy (FT-IR) spectra of UV-treated thin-film ZnO NCs under a vacuum atmosphere. The 4,000–2,000 cm^-1^ range was checked to confirm the organic ligand region. The CH_2_ absorption was confirmed at 2,800 cm^-1^ [S2]. The CH_2_ absorption decreased as the duration of UV treatment increased. Figures S4(b) and S4(c) show energy dispersive X-ray spectroscopy (EDX) spectra of UV-treated thin-film ZnO NCs. The diminished carbon intensity was confirmed.

Table S1. Chemical-composition change of UV-treated ZnO NCs under a vacuum atmosphere

| Irradiation time | C at% | O at% | Zn at% |
| --- | --- | --- | --- |
| 0 min | 35 | 32 | 33 |
| 1 min | 32 | 33 | 35 |
| 3 min | 25 | 37 | 38 |
| 5 min | 18 | 40 | 42 |
| 15 min | 10 | 44 | 46 |
| 30 min | 9 | 46 | 47 |

Table S1 presents the chemical-composition change of UV-treated ZnO NCs under a vacuum atmosphere using EDX spectra. Only the decomposition of carbon was confirmed, maintaining the Zn/O ratio after the UV treatment.

**UV irradiation under nitrogen atmosphere**

(a)

(b)

(c)

Figure S5. (a) Narrow FT-IR spectra of UV-treated thin-film ZnO NCs under a nitrogen atmosphere, EDX spectrum of UV-treated thin-film ZnO NCs under a nitrogen atmosphere: (b) 0 min and (c) 30 min.

Figure S5(a) presents FT-IR spectra of UV-treated thin-film ZnO NCs under a nitrogen atmosphere. The 4,000–2,000 cm^-1^ range was checked to confirm the organic ligand region. The CH_2_ absorption was confirmed at 2,800 cm^-1^ [S2]. The CH_2_ absorption decreased as the duration of UV treatment increased. Figures S5(b) and S5(c) show EDX spectra of UV-treated thin-film ZnO NCs. The diminished carbon intensity was confirmed.

Table S2. Chemical-composition change of UV-treated ZnO NCs under a nitrogen atmosphere

| Irradiation time | C at% | O at% | Zn at% |
| --- | --- | --- | --- |
| 0 min | 35 | 32 | 33 |
| 1 min | 31 | 34 | 35 |
| 3 min | 23 | 38 | 39 |
| 5 min | 16 | 42 | 42 |
| 15 min | 8 | 45 | 47 |
| 30 min | 6 | 46 | 48 |

Table S2 presents the chemical-composition change of UV-treated ZnO NCs under a nitrogen atmosphere according to EDX spectra. Only the decomposition of carbon was confirmed, maintaining the Zn/O ratio after the UV treatment.

**UV irradiation under air atmosphere**

(a)

(b)

(c)

Figure S6. (a) Narrow FT-IR spectra of UV-treated thin-film ZnO NCs under an air atmosphere and EDX spectrum of UV-treated thin-film ZnO NCs under an air atmosphere: (b) 0 min and (c) 30 min.

Figure S6(a) presents the FT-IR spectra of UV-treated thin-film ZnO NCs under an air atmosphere. The 4,000–2,000 cm^-1^ range was checked to confirm the organic ligand region. The CH_2_ absorption was confirmed at 2,800 cm^-1^ [S2]. The CH_2_ absorption decreased as the duration of UV treatment increased. Figures S6(b) and S6(c) show EDX spectra of the UV-treated thin-film ZnO NCs. The diminished carbon intensity was confirmed.

Table S3. Chemical-composition change of UV-treated ZnO NCs under an air atmosphere

| Irradiation time | C at% | O at% | Zn at% |
| --- | --- | --- | --- |
| 0 min | 35 | 32 | 33 |
| 1 min | 29 | 35 | 36 |
| 3 min | 19 | 40 | 41 |
| 5 min | 10 | 44 | 46 |
| 15 min | 8 | 45 | 47 |
| 30 min | 7 | 46 | 47 |

Table S3 presents the chemical-composition change of UV-treated ZnO NCs under an air atmosphere using EDX spectra. Only the decomposition of carbon was confirmed, maintaining the Zn/O ratio after the UV treatment.

**UV irradiation under oxygen atmosphere**

(a)

(b)

(c)

Figure S7. (a) Narrow FT-IR spectra of UV-treated thin-film ZnO NCs under an oxygen atmosphere, EDX spectrum of UV-treated thin-film ZnO NCs under an oxygen atmosphere: (b) 0 min and (c) 30 min.

Figure S7(a) presents the FT-IR spectra of UV-treated thin-film ZnO NCs under an oxygen atmosphere. The 4,000–2,000 cm^-1^ range was checked to confirm the organic ligand region. The CH_2_ absorption was confirmed at 2,800 cm^-1^ [S2]. The CH_2_ absorption decreased as the duration of UV treatment increased. Figures S7(b) and S7(c) show EDX spectra of the UV-treated thin-film ZnO NCs. The diminished carbon intensity was confirmed.

Table S4. Chemical-composition change of UV-treated ZnO NCs under an air atmosphere

| Irradiation time | C at% | O at% | Zn at% |
| --- | --- | --- | --- |
| 0 min | 35 | 32 | 33 |
| 1 min | 17 | 41 | 42 |
| 3 min | 12 | 43 | 45 |
| 5 min | 7 | 47 | 46 |
| 15 min | 6 | 46 | 47 |
| 30 min | 6 | 45 | 48 |

Table S4 presents the chemical-composition change of UV-treated ZnO NCs under an oxygen atmosphere according to EDX spectra. Only the decomposition of carbon was confirmed, maintaining the Zn/O ratio after the UV treatment. The remarkable carbon decomposition rate was confirmed under the oxygen atmosphere because of the abundant ozone generation.

**Distribution of ZnO NCs**

Figure S8. Optical images of ZnO NCs solution in ethanol (a) immediately after dispersion and (b) after 30 days.


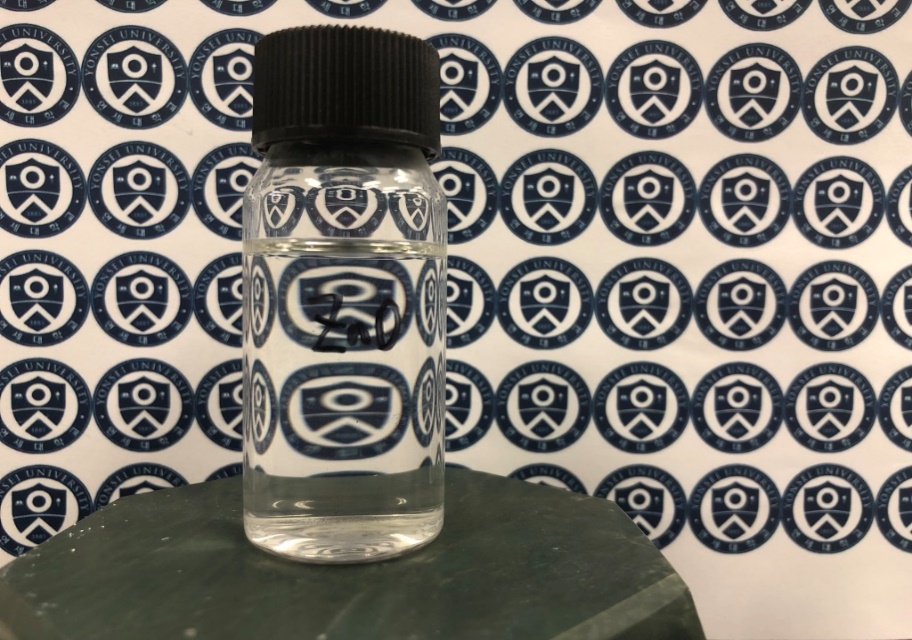

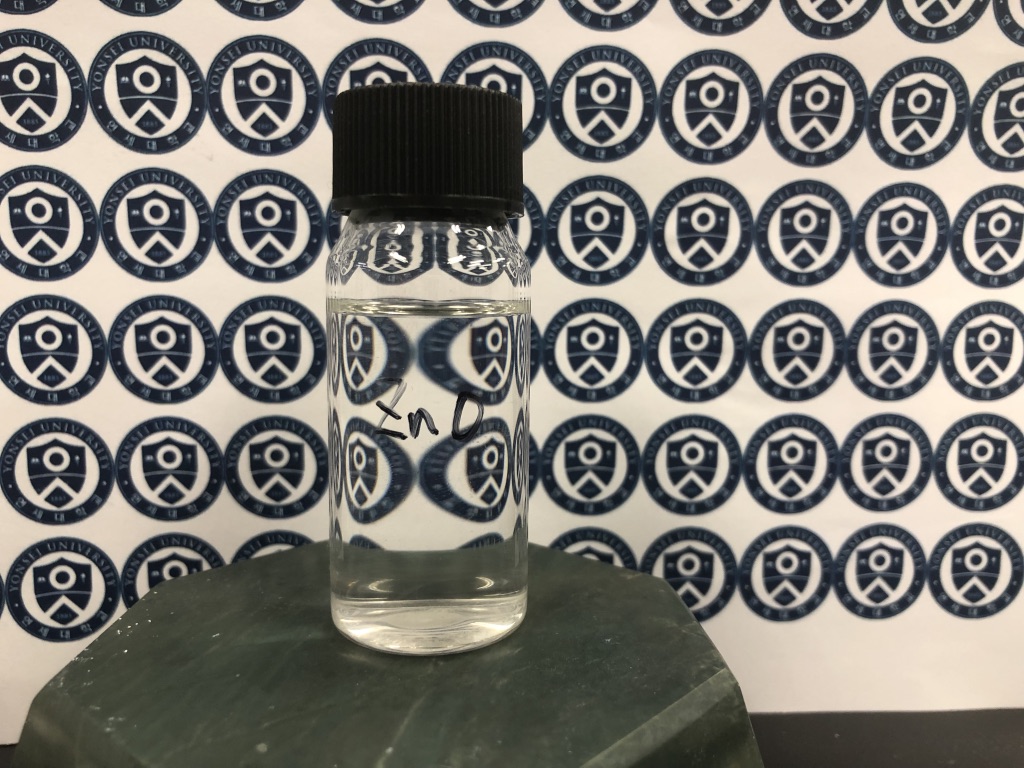


(a)

(b)

The ZnO NCs were dispersed in ethanol to form a ZnO NC thin films. The below images show as clear solution which containing the ZnO NCs. The clear solution can maintain more than 30 days. This image was added to the supplementary. The TEM image on figure 1(d) was measured using carbon Cu grid sampling from that clear solution. It can be seen that very homogeneous ZnO NCs are dispersed.


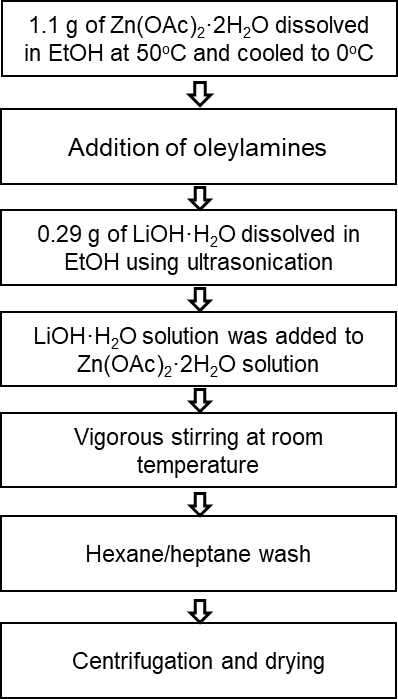
**Synthesis of ZnO nanocrystals (NCs)**

Figure S9. Flowchart of the synthesis of ZnO nanocrystals.

**UV treatment system**
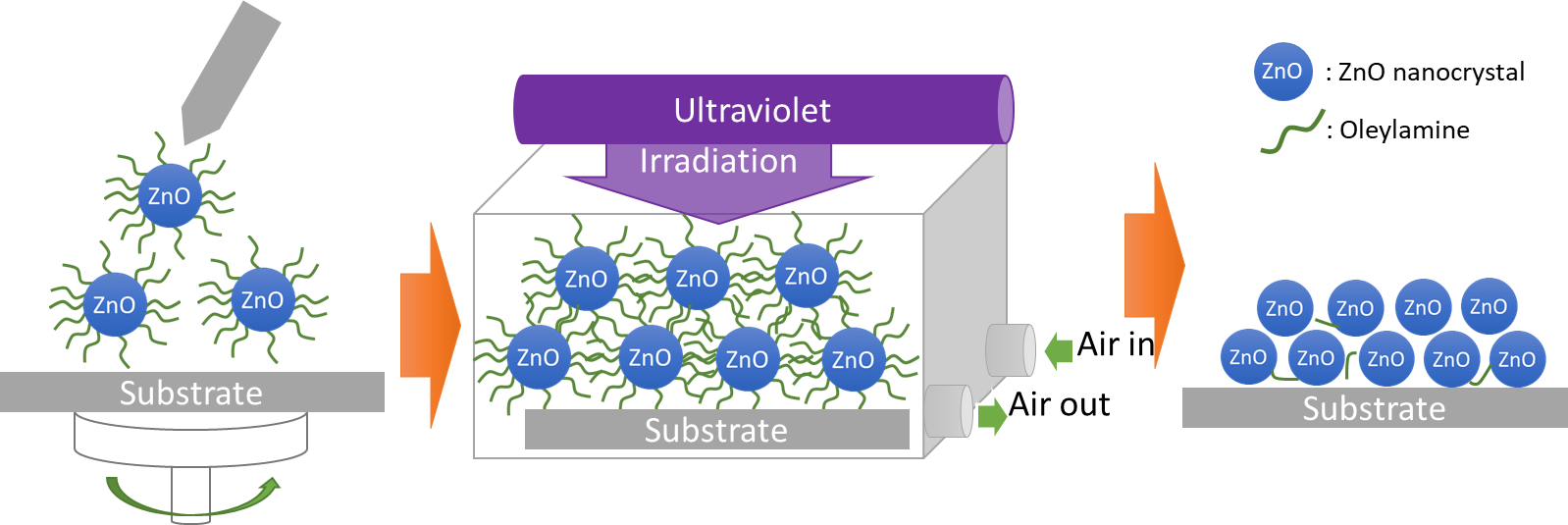


Figure S10. Schematic of the UV treatment.

**REFERENCES**

1. H. Kalita, J. Mohapatra, L. Pradhan, A. Mitra, D. Bahadur and M. Aslam, Efficient synthesis of rice based graphene quantum dots and their fluorescent properties, RSC Adv., 6 (2016) 23518-23524.
2. M. Salavati-Niasari, Z. Fereshtch, F. Daver, Synthesis of oleylamine capped copper nanocrystals via thermal reduction of a new precursor, Polyhedron, 28 (1) (2009) 126-130.
